# Successful receptor-mediated radiation therapy of xenografted human midgut carcinoid tumour

**DOI:** 10.1038/sj.bjc.6602845

**Published:** 2005-10-25

**Authors:** L Kölby, P Bernhardt, V Johanson, A Schmitt, H Ahlman, E Forssell-Aronsson, H Mäcke, O Nilsson

**Affiliations:** 1 Department of Surgery, Lundberg Laboratory for Cancer Research, Institute for Surgical Sciences, Göteborg University, Sahlgrenska University Hospital, Göteborg SE-413 45, Sweden; 2 Department of Radiation Physics, Lundberg Laboratory for Cancer Research, Göteborg University, Sahlgrenska University Hospital, Göteborg, Sweden; 3 Division of Radiological Chemistry, University Hospital, Basel, Switzerland; 4 Departments of Pathology, Lundberg Laboratory for Cancer Research, Göteborg University, Sahlgrenska University Hospital, Göteborg, Sweden

**Keywords:** carcinoid, GOT1, somatostatin receptors, [^177^Lu-DOTA^0^-Tyr^3^]-octreotate, therapy

## Abstract

Somatostatin receptor (sstr)-mediated radiation therapy is a new therapeutic modality for neuroendocrine (NE) tumours. High expression of sstr in NE tumours leads to tumour-specific uptake of radiolabelled somatostatin analogues and high absorbed doses. In this study, we present the first optimised radiation therapy via sstr using [^177^Lu-DOTA^0^-Tyr^3^]-octreotate given to nude mice xenografted with the human midgut carcinoid GOT1. The tumours in 22 out of 23 animals given therapeutic amounts showed dose-dependent, rapid complete remission. The diagnostic amount (0.5 MBq [^177^Lu-DOTA^0^-Tyr^3^]-octreotate) did not influence tumour growth and was rapidly excreted. In contrast, the therapeutic amount (30 MBq [^177^Lu-DOTA^0^-Tyr^3^]-octreotate) induced rapid tumour regression and entrapment of ^177^Lu so that the activity concentration of ^177^Lu remained high, 7 and 13 days after injection. The entrapment phenomenon increased the absorbed dose to tumours from 1.6 to 4.0 Gy MBq^−1^ and the tumours in animals treated with 30 MBq received 120 Gy. Therapeutic amounts of [^177^Lu-DOTA^0^-Tyr^3^]-octreotate rapidly induced apoptosis and gradual development of fibrosis in grafted tumours. In conclusion, human midgut carcinoid xenografts can be cured by receptor-mediated radiation therapy by optimising the uptake of radioligand and taking advantage of the favourable change in biokinetics induced by entrapment of radionuclide in the tumours.

Midgut carcinoids are the most frequent neuroendocrine (NE) tumours of the gastrointestinal tract and often present with widespread disease and severe hormonal symptoms (Modlin *et al*, 2003). These tumours have high expression of somatostatin receptors (sstr), which can be exploited for diagnostic and therapeutic purposes (Nilsson *et al*, 1998). The most sensitive method to detect carcinoid metastases is sstr scintigraphy using ^111^In coupled with the somatostatin analogue octreotide via the chelator DTPA (Krenning *et al*, 1993; Ahlman *et al*, 1994; Cimitan *et al*, 2003). Pharmacological treatment of the carcinoid syndrome with long-acting somatostatin analogues reduces hormone release and related symptoms in the majority of patients. However, there is no evidence that somatostatin analogues exert antiproliferative actions on these tumours, although individual cases with induction of apoptosis have been reported (Eriksson and Öberg, 1999; Öberg, 1999; Bousquet *et al*, 2001). The only curative treatment for carcinoid tumours is surgical, but due to late diagnosis, radical surgery is seldom possible. Debulking procedures offer palliation of hormonal symptoms and may prolong survival (McEntee *et al*, 1990; Ahlman *et al*, 2000). Palliative treatment may include chemotherapy and biotherapy with interferon, both with limited benefits and adverse effects (Öberg, 1993; Moertel *et al*, 1994; Kölby *et al*, 2003). Following interventional and medical treatment, the 5-year survival is as high as 69% for patients with liver metastases (Wängberg *et al*, 1996; Hellman *et al*, 2002).

A new treatment strategy is directed radiation therapy with radiolabelled somatostatin analogues. The first therapies were attempted with [^111^In-DTPA^0^]-octreotide and resulted in reduced tumour markers such as chromogranin A (CgA, the general NE marker) and 5-hydroxy indole acetic acid (5-HIAA, the serotonin metabolite), but only occasionally objective tumour responses (Fjälling *et al*, 1996; McCarthy *et al*, 1998; Krenning *et al*, 1999). ^111^In emits mainly photons that will cause a high whole body irradiation, which limits its use for therapy (Bernhardt *et al*, 2001). ^111^In also emits Auger electrons with extremely short range with the potential to cause cell death, if the radionuclide can be allocated to the nucleus (Bernhardt *et al*, 2003b). Using the high-energy beta emitter ^90^Y, coupled to [Tyr^3^]-octreotide via the chelator DOTA (1,4,7,10-tetraazacyclododecane,1,4,7,10-tetraacetic acid), partial tumour remission was achieved in 7–24% of the patients (Paganelli *et al*, 2001; Waldherr *et al*, 2001; Valkema *et al*, 2002). Development of new somatostatin analogues with higher receptor affinity and higher degree of receptor internalisation will improve the therapeutic efficacy. The new somatostatin-based radioligand [DOTA^0^,Tyr^3^,Thr^8^]-octreotide (=[DOTA^0^,Tyr^3^]-octreotate) has a higher affinity for sstr2 than [DOTA^0^,Tyr^3^]-octreotide, and coupled with the medium-energy *β*-emitter ^177^Lu, high absorbed doses can be achieved (de Jong *et al*, 1997; Reubi *et al*, 2000; de Jong *et al*, 2001). ^177^Lu has a shorter range than ^90^Y and is therefore better suited for therapy of smaller tumours (Bernhardt *et al*, 2001; Bernhardt *et al*, 2003a). Previous experimental studies with xenografts have been performed using rat pancreatic acinar tumours carrying sstr, while the GOT1 model is an authentic human carcinoid with preserved phenotype (de Jong *et al*, 1998, 2001; Kölby *et al*, 2001). In a recent series, patients with large NE tumour burden were treated with [^177^Lu-DOTA^0^-Tyr^3^]-octreotate and partial tumour regression was seen in 35% and complete remission in 3% (Kwekkeboom *et al*, 2003).

In the present study, we analysed the effect of treatment with [^177^Lu-DOTA^0^-Tyr^3^]-octreotate of the human midgut carcinoid GOT1 xenografted to nude mice. Our aims were to optimise the uptake of [^177^Lu-DOTA^0^-Tyr^3^]-octreotate by defining the point of receptor saturation following a single i.v. injection of the radiopharmaceutical and to evaluate the effect of sstr-mediated radiation therapy with [^177^Lu-DOTA^0^-Tyr^3^]-octreotate given to xenografted midgut carcinoid tumours.

## MATERIAL AND METHODS

### Animal model

The transplantable human midgut carcinoid GOT1 was established as previously described (Kölby *et al*, 2001). In brief, cultured GOT1 cells were inoculated subcutaneously into nude mice. After 6 months, tumours became visible. These tumours were minced into pieces and new generations of tumours were generated by transplantation of tumour tissue to the subcutis of nude mice. When the tumours were 5–10 mm in size, the experiments were started. The experiments were approved by the Ethical Committee for Animal Research at Göteborg University. All animal procedures were consistent with the animal use guidelines of the UKCCCR.

### Pharmaceuticals

Kits of [DOTA^0^-Tyr^3^]-octreotate were made by mixing 2.02 mg [DOTA^0^-Tyr^3^]octreotate (synthesized by Jörg Schmitt, Division of Radiological Chemistry, University Hospital, Basel, Switzerland) with 630 mg sodium ascorbate (VWR International AB, Sweden), 125 mg gentisic acid (2,5-dihydroxy benzoic acid; VWR International AB, Sweden) and 5 ml 0.05 M HCl. Each kit contained 40 *μ*g of [DOTA^0^-Tyr^3^]-octreotate. The compound was stored at −25°C until use. ^177^LuCl_3_ (Perkin Elmer, Life Sciences, Inc., Boston, MA, USA) was diluted in 0.05 M HCl and mixed with [DOTA^0^-Tyr^3^]-octreotate to a specific activity of 30 MBq *μ*g^−1^. For the biokinetic studies with 0.5 MBq [^177^Lu-DOTA^0^-Tyr^3^]-octreotate, the specific activity was 0.5 MBq *μ*g^−1^. The mixture was incubated for 30 min at 80°C (de Jong *et al*, 2001).

[DTPA]-octreotide was labelled with ^111^In according to the manufacturer's instructions (Mallinckrodt Medical B.V., The Netherlands).

The peptide-bound fraction of ^177^Lu and ^111^In was assessed by instant thin layer chromatography (ITLC-SG, Gelman, Ann Arbor, MI, USA) with 0.1 M sodium citrate (pH=5.0, VWR International AB, Sweden) as mobile phase. The fraction of peptide-bound ^177^Lu and ^111^In was more than 99%.

The exact amount of radiopharmaceutical administered was determined by measuring the activity in the syringes before and after administration of the radiopharmaceutical with a well-type ionisation chamber (CRC-15R, Capintec, NJ, USA).

### Biodistribution and biokinetics of [^177^Lu-DOTA^0^-Tyr^3^]-octreotate

For studies of the biodistribution of [^177^Lu-DOTA^0^-Tyr^3^]-octreotate, five groups of tumour-bearing animals were injected with a single i.v. injection of 7.5, 15, 30, 60 and 120 MBq of ^177^Lu-DOTA-Tyr^3^-octreotate (30 MBq *μ*g^−1^), respectively (*n*=5 in all groups). The animals were killed 24 h later and tumour tissue and multiple organs were collected for measurement of radioactivity and histopathological analyses. The ^177^Lu activity was measured using a Wallac 1480 gamma counter (WIZARD®3″, Wallac, Oy, Finland). Correction was made for detector background and radioactive decay. The activity concentration (*C*) of the radionuclide was expressed as the fraction of injected activity per unit mass of the tissue (%IA g^−1^).

For studies of biokinetics, tumour-bearing animals were injected with 0.5 MBq (1 *μ*g, *n*=20) or 30 MBq (1 *μ*g, *n*=20) [^177^Lu-DOTA^0^-Tyr^3^]-octreotate, that is, diagnostic and therapeutic amounts of radionuclide, respectively. All animals were injected with a single i.v. injection and were killed in groups of five at 1, 3, 7 and 13 days post injection (p.i.), respectively. Control animals (*n*=5) were untreated. Tumour tissue and multiple organs were collected for measurements of radionuclide uptake and histopathological analyses.

The activity concentration (%IA g^−1^) was determined for tumour tissue (*C*_T_) and normal tissue (*C*_N_). The tumour-to-normal tissue activity concentration ratio (TNC) was calculated according to the formula TNC_N_=*C*_T_/*C*_N_.

### Dosimetry

The calculation of the absorbed dose to tumours, kidneys, liver and bone marrow was based on the pharmacokinetics of 0.5 and 30 MBq [^177^Lu-DOTA^0^-Tyr^3^]-octreotate (Loevinger, 1988). The cumulative activity concentration, that is, the total number of radioactive decays per mass unit, was determined by estimating the area under the curve for the activity concentration in each organ *vs* time. The electron energy emitted per decay was assumed to be 147 keV (Sowby, 1983). The contribution from photons was neglected. Also, the contribution from organs other than the target organ was neglected.

### Therapeutic effects of ^177^Lu-DOTA-Tyr^3^-octreotate

The therapeutic effect of ^177^Lu-octreotate was studied in five groups of tumour-bearing animals given 7.5 (0.25 *μ*g, *n*=6), 15 (0.5 *μ*g, *n*=6), 30 (1 *μ*g, *n*=6), 60 (2 *μ*g, *n*=7) or 120 (4 *μ*g, *n*=10) MBq of [^177^Lu-DOTA^0^-Tyr^3^]-octreotate, respectively. Control animals (*n*=11) were given 4 *μ*g of [DOTA-Tyr^3^]-octreotate. Tumour size was measured and the volume was calculated according to the formula *V*=4*πab*^2^/3 (*a* is the longest radius and *b* is the transverse radius). The animals were followed for 2–7 months. Complete response (CR) was defined as >99% reduction of tumour volume. Partial response (PR) was defined as 50–99% reduction and minor response (MR) as <50% reduction of tumour volume.

### Biodistribution of [^111^In-DTPA]-octreotide

In order to make it possible to compare the uptake of radiolabelled somatostatin analogue with previous clinical studies, the binding of radiolabelled octreotide was investigated. Each animal (*n*=5) was injected i.v. with 4 MBq (0.2 *μ*g) of [^111^In-DTPA]-octreotide 24 h before killing. Tumours and blood samples were collected and weighed, and the ^111^In activity in tumour and blood was measured using the gamma counter. The TNC_Blood_ was calculated as described above (Forssell-Aronsson *et al*, 1995).

### Morphological analysis

Morphological analysis was carried out on all tumours included in this study to verify potential therapeutic effects. Tumour tissue from all animals were harvested and fixed in 4% paraformaldehyde (PF) in PBS at pH 7.4 for 24 h. Specimens were subsequently dehydrated and embedded in paraffin wax. Parallel sections were counterstained with haematoxylin (htx) and eosin for morphological analysis, and with Ladewig staining to facilitate the detection of fibrin thrombi in tumour vessels. In all animals given a therapeutic amount of [^177^Lu-DOTA^0^-Tyr^3^]-octreotate, microscopic examination of liver, kidneys and bone marrow was performed in order to reveal any morphological changes related to radiation. Deparaffinised sections were preincubated with 5% nonfat dry milk followed by incubation overnight with primary antibodies, listed in Table 1. Antiserum was diluted in PBS containing 1% BSA and 0.1% sodium azide. Bound antibodies were visualised by the indirect immunoperoxidase technique (EnVision™+, cat. no. K4000, Dakopatts, Glostrup, Denmark).

### Apoptotic cell count

The number of apoptotic tumour cells was investigated in tumour tissue from the biokinetic study (animals injected with 30 MBq ^177^Lu-octreotate and followed up to 13 days). Haematoxylin–eosin-stained sections were used to identify apoptotic tumour cells according to morphological criteria (condensed and fragmented nuclei and eosinophilic cytoplasma). Three high power fields (HPF; × 40 objective) were selected from each tumour for counting of apoptotic cells. To obtain representative areas of the tumour, a direction from the centre to the periphery of the tumour was randomly selected. Photographs of each HPF were then systematically taken: (i) in the centre, (ii) half way to the periphery and (iii) in the periphery of the tumour, all along the selected line. The number of apoptotic cells was counted on the photographs and the average number of apoptotic cells per HPF calculated (apoptotic cell count).

### Statistical analysis

Differences between groups regarding uptake of [^177^Lu-DOTA^0^-Tyr^3^]-octreotate, TNC value and apoptotic cell count were analysed by one-way analysis of variance. Logarithmic transformation was used for variance stabilisation. Compensation for multiple comparisons was performed using the Dunnett's method (Hochberg and Tamane, 1987).

Differences in biokinetics between 30 and 0.5 MBq were analysed by two-way analysis of variance followed by *t*-test at each time point. Logarithmic transformation was used for variance stabilisation. Compensation for mass significance was performed according to Bonferroni-Holm (Hochberg and Tamane, 1987).

For analysis of the therapeutic effect of [^177^Lu-DOTA^0^-Tyr^3^]-octreotate, the number of CR in the treatment groups (7.5–120 MBq) was compared to the number of CR in the control group using Fisher's exact test. Compensation for mass significance was performed according to Bonferroni-Holm.

*P*-values <0.05 were considered significant.

## RESULTS

### Biodistribution of [^177^Lu-DOTA^0^-Tyr^3^]-octreotate

Five groups of tumour-bearing animals were given 7.5, 15, 30, 60 and 120 MBq [^177^Lu-DOTA^0^-Tyr^3^]-octreotate, respectively. The maximum uptake of [^177^Lu-DOTA^0^-Tyr^3^]-octreotate in GOT1 tumours 24 h p.i. was seen both in animals given 7.5 and 15 MBq and reached 17±3% IA g^−1^ (mean±s.e.m.). In animals given larger amounts of activity, the uptake declined, indicating saturation of sstr in tumour tissue.

**Figure 1:**
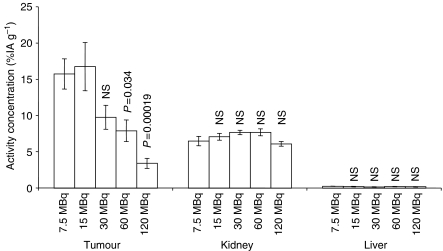
Uptake of [^177^Lu-DOTA^0^-Tyr^3^]-octreotate was studied in tumours as well as in several organs 24 h p.i. In the tumours, the maximum uptake was 17±3.3%IA g^−1^ (mean±s.e.m. *n*=5 for all groups) and was seen in animals receiving 15 MBq (0.5 *μ*g) and 7.5 MBq (0.25 *μ*g) [^177^Lu-DOTA^0^-Tyr^3^]-octreotate. Decreasing uptake for higher administered doses indicated saturation of sstr in tumour tissue. For the critical organs kidney and liver, no signs of saturation was seen. *P*-values reflect the difference *vs* 7.5 MBq.

In normal tissues, the uptake was generally lower than in tumours. The highest uptake was found in the kidneys (6.1–7.7%IA g^−1^) (Figure 1). For all other organs (liver, spleen, small intestine, blood, skeletal muscle, heart, brain, lungs, pancreas and adrenals), the uptake was very low (0.01–1.7) %IA g^−1^. For kidneys, liver, blood, skeletal muscle, heart and brain, the uptake was independent of the amount of activity administered, which indicates no saturation, or nonspecific distribution in the tissues. However, in the spleen, small intestine, lungs, pancreas and adrenals, the uptake was highest for 7.5 MBq [^177^Lu-DOTA^0^-Tyr^3^]-octreotate and declined with increasing amounts of [^177^Lu-DOTA^0^-Tyr^3^]-octreotate, which indicates saturation of receptor-mediated binding.

### Biokinetics of [^177^Lu-DOTA^0^-Tyr^3^]-octreotate

In order to compare the biokinetics of [^177^Lu-DOTA^0^-Tyr^3^]-octreotate after diagnostic and therapeutic amounts, two groups of animals were injected with 0.5 and 30 MBq [^177^Lu-DOTA^0^-Tyr^3^]-octreotate, respectively. The specific activity of [^177^Lu-DOTA^0^-Tyr^3^]-octreotate in the diagnostic group was lower, that is, the amount of peptide (1 *μ*g) was identical in both groups.

**Figure 2:**
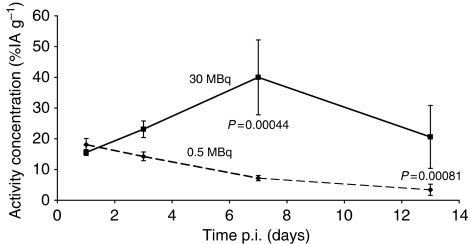
The biokinetics of [^177^Lu-DOTA^0^-Tyr^3^]-octreotate after a diagnostic amount (0.5 MBq) as well as after a therapeutic amount (30 MBq) was studied in two groups of animals injected with [^177^Lu-DOTA^0^-Tyr^3^]-octreotate. In the animals receiving 0.5 MBq [^177^Lu-DOTA^0^-Tyr^3^]-octreotate, the activity concentration declined over time. On the contrary, in animals receiving 30 MBq, the activity concentration was retained over time and the biokinetic curves differed significantly both at 7 days p.i. (*P*=0.00044) and 13 days p.i. (*P*=0.00081). The values are presented as mean±s.e.m., *n*=5 at all time points for both groups.

The uptake of [^177^Lu-DOTA^0^-Tyr^3^]-octreotate in tumours was similar for both groups of animals 24 h p.i. However, the biokinetics of the activity concentration differed significantly between the two groups. In animals given 0.5 MBq [^177^Lu-DOTA^0^-Tyr^3^]-octreotate, which did not retard tumour growth, the activity concentration declined over time and was less than 50% at 7 days and less than 20% at 13 days p.i. compared to the initial concentration. In contrast, in animals given 30 MBq, the activity concentration remained high over time and the biokinetic curves differed significantly both at 7 days p.i. (*P*=0.00044) and 13 days p.i. (*P*=0.00081) (Figure 2). The tumour volumes at 7 days p.i. and 13 days p.i. for animals given 30 MBq were reduced to 16±8.5 and 11±4.2% (mean±s.e.m.) compared to the initial volumes.

### Tumour-to-normal tissue activity concentration ratio

**Figure 3:**
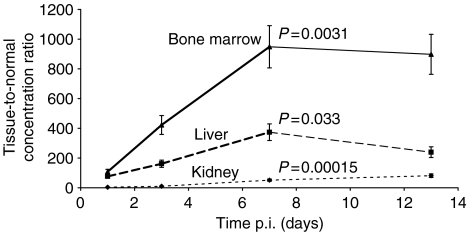
Tumour-to-normal tissue activity concentration ratio (TNC) of [^177^Lu-DOTA^0^-Tyr^3^]-octreotate followed up to 13 days after a single injection of 30 MBq. At 3, 7 and 13 days p.i., the TNC value increased compared to the situation 1 day p.i. This indicates a favourable relation over time for the radiation to the tumour in comparison with the radiation to the critical organs liver, kidney and bone marrow. *P*-values reflect the difference *vs* 1 day p.i.

Tumour-to-normal tissue activity concentration ratios of [^177^Lu-DOTA^0^-Tyr^3^]-octreotate were determined up to 13 days after a single injection of 30 MBq. In general, the TNC value increased compared to the situation at 24 h p.i., which indicates a favourable change over time for the radiation to the tumour in relation to the radiation received by liver, kidneys and bone marrow. The increase in TNC value at 7 days p.i., compared to 24 h p.i., was significant for the bone marrow (*P*=0.0031), liver (*P*=0.033) as well as for the kidneys (*P*=0.00015) (Figure 3).

### Dosimetry

Tumours treated with 30 MBq received about 120 Gy, corresponding to 4.0 Gy MBq^−1^, when the absorbed dose was calculated using biokinetic data for the therapeutic amount of 30 MBq. For normal tissues in animals given 30 MBq, the absorbed doses were 0.15 Gy to bone marrow, 0.68 Gy to liver and 8.7 Gy to kidneys, respectively. The diagnostic amount 0.5 MBq, with clearly different biokinetics, resulted in 1.6 Gy MBq^−1^ to the tumours.

### Therapeutic effects of [^177^Lu-DOTA^0^-Tyr^3^]-octreotate

**Figure 4:**
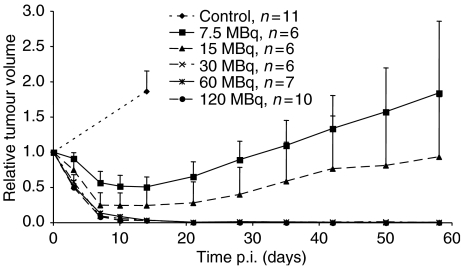
Treatment with [^177^Lu-DOTA^0^-Tyr^3^]-octreotate resulted in a rapid, dose-dependent reduction of tumour volume. For animals receiving 7.5 and 15 MBq, the effect was moderate and temporary, whereas for animals receiving 30 MBq CR was achieved. In animals treated with 30, 60 or 120 MBq, 22 out of 23 animals had CR. The tumour volume presented is the mean volume±s.e.m. for each group, set to 1 at the start of the experiment. The *P*-values for the number of CR for animals treated with 30, 60 and 120 MBq *vs* controls are 0.00032, 0.0011 and 0.000014, respectively.

Five groups of tumour-bearing animals were injected with 7.5, 15, 30, 60 and 120 MBq ^177^Lu-DOTA-Tyr^3^-octreotate, respectively. Control animals were given 4 *μ*g of unlabelled DOTA-Tyr^3^-octreotate. The tumours in control animals grew rapidly and doubled their volumes after 2 weeks. Treatment with [^177^Lu-DOTA^0^-Tyr^3^]-octreotate resulted in a rapid and dose-dependent reduction of tumour volume (Figure 4). The number of CR increased significantly with increasing amounts of [^177^Lu-DOTA^0^-Tyr^3^]-octreotate administered (Table 2).

After treatment with 30 MBq, one out of six animals with CR remained free of recurrent tumour until being killed 3 months later. After 60 MBq, two out of six animals with CR remained free of recurrence until being killed 6 –7 months later. After treatment with 120 MBq, six out of 10 animals with CR remained free of recurrence until being killed 3–4 months after the remission (Figure 4).

### Biodistribution of [^111^In-DTPA]-octreotide

The activity concentration of [^111^In-DTPA]-octreotide in GOT1 tumours 24 h p.i. was 8.2±2.0%IA g^−1^ (mean±s.e.m.). The TNC_Blood_ for [^111^In-DTPA]-octreotide was then 410±70 (mean±s.e.m.).

### Induction of apoptosis and necrosis after treatment with [^177^Lu-DOTA^0^-Tyr^3^]-octreotate

**Figure 5:**
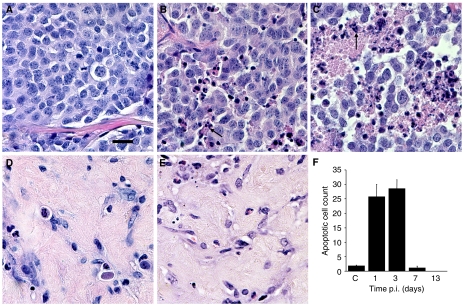
Typical morphological appearance of GOT1 tumours 1 day (**B**), 3 days (**C**), 7 days (**D**) and 13 day (**E**) after treatment with 30 MBq [^177^Lu-DOTA^0^-Tyr^3^]-octreotate compared to an untreated tumour (**A**). After injection of [^177^Lu-DOTA^0^-Tyr^3^]-octreotate, significant increases in the apoptotic cell count *vs* controls could be seen both 1 day p.i. (*P*=0.0000030) and 3 day p.i. (*P*=0.00000059). At 3 days p.i., all five tumours presented with large confluent necroses and oedema. At 7 and 13 days p.i., the apoptotic cell count decreased and the oedema and necroses were gradually replaced by fibrosis. The apoptotic cell count was 1.9±0.29 (mean±s.e.m.) for control animals and 26±4.3, 29±3.1, 1.2±0.65 and 0±0 at 1, 3, 7 and 13 days p.i., respectively (**F**). Typically, apoptotic cells (condensed and fragmented nuclei and eosinophilic cytoplasma) are indicated by arrows. Scale bar equals 20 *μ*m.

In untreated controls, tumour cells grew in solid sheets with small variation in nuclear size and without necroses. The tumour cells were positive for the general NE-marker CgA, serotonin (5-HT) and Vesicular Mono Amine Transporters (VMAT1 and 2). In animals treated with 30 MBq [^177^Lu-DOTA^0^-Tyr^3^]-octreotate, a significant increase in apoptotic cell count, compared to controls, was observed both at 1 day p.i. (*P*=0.0000030) and 3 days p.i. (*P*=0.00000059). In animals killed 3 days p.i., all five tumours had large confluent necroses and intercellular oedema. In animals, killed 7 and 13 days p.i., the number of tumour cells was clearly reduced as was the apoptotic cell count and the oedema and necroses were replaced by fibrosis. The typical appearance of a tumour 13 days p.i. was that of almost complete fibrosis and absence of tumour cells (Figure 5). The apoptotic cell count at each time point is presented in Figure 5F.

**Figure 6:**
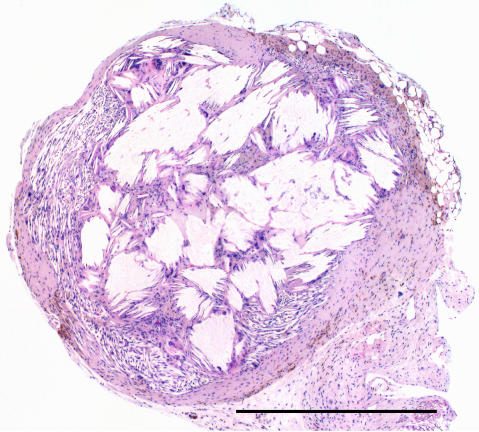
Histopathological analysis of a small remnant of tumour tissue in an animal with CR 5 months after administration of 120 MBq [^177^Lu-DOTA^0^-Tyr^3^]-octreotate. The tumour residue consisted of a brownish nodule (2 mm in diameter). In the periphery, only a thin rim of fibroblasts was found. The dominant part of the nodule contained crystalline structures surrounded by inflammatory cells including macrophages and giant cells. Scale bar equals 1 mm.

In the therapy groups (7.5–120 MBq), all tumours, or tumour residues, were morphologically examined at the end of the observation period. Gross examination of the small residual tumour tissue, found in animals with CR in response to [^177^Lu-DOTA^0^-Tyr^3^]-octreotate, revealed brownish nodules, 1–2 mm in diameter. The microscopic analysis demonstrated a thin rim of fibroblasts in the periphery and a central part consisting of crystalline structures surrounded by inflammatory cells including macrophages and giant cells (Figure 6). Specific staining for fibrin (Ladewig) did not show any signs of thrombosis in treated tumours.

### Side effects of radiation

Morphologic analysis of the liver, kidneys and bone marrow after [^177^Lu-DOTA^0^-Tyr^3^]-octreotate therapy revealed two animals with reactive inflammatory response in the portal fields of the liver. These inflammatory infiltrates were composed both of CD 3-positive T cells and CD 20-positive B cells and had a Ki-67 proliferative index of <1%. In one animal, the kidney contained a fibrous scar. The bone marrow of all animals was normal with preserved haematopoietic cells.

## DISCUSSION

This study presents the first systematic optimisation of sstr-mediated radiation therapy with [^177^Lu-DOTA^0^-Tyr^3^]-octreotate of a human midgut carcinoid xenografted to nude mice. The first part of the study demonstrated that saturation of sstr in the tumours after a single i.v injection of [^177^Lu-DOTA^0^-Tyr^3^]-octreotate was evident between 0.5 *μ*g (15 MBq) and 1 *μ*g (30 MBq). It is important to define this level, since suboptimal therapeutic amounts will not lead to maximal uptake and maximal absorbed doses in the tumours. The uptake, and hence the absorbed dose, in the kidneys, liver and bone marrow increased proportionally to the amounts administered. Therefore, amounts in excess of the tumour saturation level will increase adverse effects of radiation therapy.

The second part of the study described the markedly different biokinetics of [^177^Lu-DOTA^0^-Tyr^3^]-octreotate, given in diagnostic *vs* therapeutic amounts. The activity concentration in tumours after being given diagnostic amounts (0.5 MBq, 1 *μ*g) was reduced over time. On the other hand, in tumours from animals given a therapeutic amount (30 MBq, 1 *μ*g), the concentration remained high over time and significantly differed from the animals given a diagnostic amount both at 7 and 13 days p.i. The preserved high activity concentration in the therapy group was unexpected and significantly contributed to the high absorbed doses in the tumours, and thereby a very high efficacy of the radiation therapy. The difference in biokinetics can to a large extent be explained by tumour shrinkage, but other factors may also be of importance, for example, upregulation of sstr expression, radionuclide sequestration, increased intratumoural pressure and changed tumour blood flow initiated by the radiation.

The entrapment of radionuclide and altered biokinetics in the tumours after a therapeutic amount resulted in significantly increased TNC values over time. Therefore, choice of a radionuclide with half-life long enough to take advantage of this favourable change is essential when sstr-mediated radiation therapy is planned.

The third part of the study showed dose-dependent tumour responses. In animals treated with 7.5 and 15 MBq [^177^Lu-DOTA^0^-Tyr^3^]-octreotate, the absorbed doses were low and CR was seen only occasionally. These tumours started to grow again after 2 weeks of reduced volume. In animals treated with 30–120 MBq [^177^Lu-DOTA^0^-Tyr^3^]-octreotate, the absorbed doses were high; all, but one, of these animals showed CR. It is very unusual to obtain CR in animal tumour models using radionuclide therapy. Besides the present study and our previous study on [^177^Lu-DOTA^0^-Tyr^3^]-octreotate therapy in the human small-cell lung cancer cell line NCI-H69 xenografted to nude mice, CR is very unusual (de Jong *et al*, 2001; Schmitt *et al*, 2004). For a long period, carcinoid tumours were considered to be resistant to radiation therapy. However, a palliative role for both external radiation and ^131^I-MIBG radiation therapy of NE tumours was proposed some 10 years ago (Samlowski *et al*, 1986; Kimmig, 1994). The present study clearly shows that human midgut carcinoid xenografts can be cured by adequate radiation therapy with minimal adverse effects.

Sstr-mediated radiation therapy using [^177^Lu-DOTA^0^-Tyr^3^]-octreotate in clinical series of patients with large tumour burden has not resulted in high rates of CR, most probably due to the administration of suboptimal amounts of radiopharmaceutical (Kwekkeboom *et al*, 2003). Our experiments show the importance of defining the amount of peptide required to achieve receptor saturation. This should, therefore, be included as part of the dose planning before therapy. Also correct choice of radionuclide and somatostatin analogue is of importance to obtain a high absorbed dose, which is a pre-requisite for a good tumour response (Bernhardt *et al*, 2001; Bernhardt *et al*, 2003a).

The present study not only showed a high cure rate of human midgut carcinoids but also revealed an unexpected entrapment of ^177^Lu in tumour tissue during therapy. As recently shown in a patient with limited lymphoglandular spread of midgut carcinoid tumour, [^177^Lu-DOTA^0^-Tyr^3^]-octreotate has the potential to induce almost complete remission (Ahlman *et al*, 2004). GOT1 cells were harvested from the first patient, who underwent therapy with [^111^In-DTPA]-octreotide. The biological characteristics of GOT1 closely resemble the tumour of the original patient, that is, preserved neuroendocrine differentiation and molecular markers, preserved sstr expression and a TNC_Blood_ value of about 400 for [^111^In-DTPA]-octreotide uptake (Fjälling *et al*, 1996; Kölby *et al*, 2001). In other aspects, that is, subcutaneous tumour localisation and more rapid tumour growth, the model differs from the human situation. An orthotopic model, with liver metastases, would be very helpful to further resemble the clinical situation. The exceptional results obtained in GOT1 xenografts may thus indicate a possibility for more successful treatment of patients with metastatic carcinoid tumours than hitherto achieved in clinical series. In the individual patient, determination of sstr expression profiles in tumours by real-time quantitative PCR may be helpful to select the best somatostatin analogue to obtain maximal uptake of the radiopharmaceutical. Also, a radionuclide with suitable range in relation to the volumes of the tumours intended to be treated is important (Bernhardt *et al*, 2001). Determination of the specific uptake in tumours (studied in biopsies or determined by octreotide scintigraphy) can also be helpful to estimate the absorbed dose and expected biological effect (Forssell-Aronsson *et al*, 1995). In the future, the dose dependency of the shown entrapment as well as the tolerance of normal tissues to sstr-mediated radiation therapy must be studied in detail before this type of treatment can be transferred to the clinical situation with high success rate.

## Figures and Tables

**Table 1:** Primary antibodies used for immunocytochemistry

**Antibody**	**Dilution**	**Clone**	**Cat. no**	**Company**
CgA (monoclonal, anti-human)	1 : 1000	LK2H10	1199021	Boehringer Mannheim, Mannheim, Germany
Ki-67 (monoclonal, anti-human)	1 : 100	MIB-1		DAKO, Glostrup, Denmark
CD 3 (monoclonal, anti-human)	1 : 100		M7254	DAKO, Glostrup, Denmark
CD 20 (M-20, goat anti-mouse)	1 : 50		Sc-7735	Santa Cruz Biotechnology, Inc., Santa Cruz, CA, USA

**Table 2:** Result of therapy with [^177^Lu-DOTA^0^-Tyr^3^]-octreotate

**Administered amount of ^177^Lu-DOTA-Tyr^3^-octreotate**	**Complete response (CR), >99% reduction of tumour volume, *P***-**value *vs* controls**	**Partial response (PR) 50–99% reduction of tumour volume**	**Minor response (MR) <50% reduction of tumour volume**
0 MBq, *n*=11 (controls)	0	0	0
			
7.5 MBq, *n*=6	0 NS	3	3 (33–46% reduction)
15 MBq, *n*=6	2 NS	3	1 (49% reduction)
30 MBq, *n*=6	6 *P*=0.00032	—	—
60 MBq, *n*=7	6 *P*=0.0011	1 (98% reduction)	—
120 MBq, *n*=10	10 *P*=0.000014		—

NS=nonsignificant.
